# Construction of a nomogram model to predict arteriosclerosis in middle-aged and elderly community dwellers: insights from a cohort study

**DOI:** 10.3389/fmed.2026.1672197

**Published:** 2026-06-19

**Authors:** Wenxing Gao, Yue Zhang, Xulei Tang, Li Yan, Zuojie Luo, Guijun Qin, Lulu Chen, Qin Wan, Zhengnan Gao, Weiqing Wang, Guang Ning, Yiming Mu

**Affiliations:** 1 People’s Liberation Army Medical School, Beijing, China; 2 Department of Endocrinology, The First Medical Center of People’s Liberation Army General Hospital, Beijing, China; 3 Department of Endocrinology, The First Hospital of Lanzhou University, Lanzhou, Gansu, China; 4 Sun Yat-sen Memorial Hospital, Zhongshan University, Guangzhou, Guangdong, China; 5 Department of Endocrinology, The First Affiliated Hospital of Guangxi Medical University, Nanning, Guangxi, China; 6 Department of Endocrinology, The First Affiliated Hospital of Zhengzhou University, Zhengzhou, Henan, China; 7 Union Hospital, Tongji Medical College, Wuhan, Hubei, China; 8 Department of Endocrinology, Affiliated Hospital of Luzhou Medical College, Luzhou, Sichuan, China; 9 Department of Endocrinology, Dalian Municipal Central Hospital, Dalian, Liaoning, China; 10 Ruijin Hospital, Shanghai Jiao Tong University School of Medicine, Shanghai, China

**Keywords:** arteriosclerosis, cardiovascular diseases, hypertension, nomogram, prediction

## Abstract

**Background:**

The incidence of arteriosclerosis is steadily increasing, and arteriosclerosis is closely associated with cardiovascular diseases. The objective of this research was to create and verify a tool for forecasting arteriosclerosis in middle-aged and elderly individuals within the community.

**Methods:**

A cohort study was conducted in multiple communities, and 4107 participants over 40 years of age were enrolled. The participants were randomly divided into a derivation cohort (*n* = 2875) and a validation cohort (*n* = 1232) at a ratio of 7:3. LASSO analysis and multivariate logistic regression were employed to analyze factors influencing arteriosclerosis and to establish a prediction model, which was visualized as a nomogram and then evaluated. The primary outcome is incident arteriosclerosis, defined as baPWV ≥ 1400 cm/s.

**Results:**

Over an average follow-up period of 3.25 ± 1.14 years, 1688 subjects (41.0%) were diagnosed with arteriosclerosis. Independent risk factors for arteriosclerosis included age, BMI, hypertension, triglyceride levels, glycosylated hemoglobin, sex, and fasting blood glucose. The area under the receiver operating characteristic curve for the derivation and validation cohorts was 0.811 (95% CI: 0.795–0.827) and 0.816 (95% CI: 0.796–0.830), respectively. The Hosmer-Lemeshow test showed good model accuracy (*P* = 0.123, *P* = 0.428). Calibration curves illustrated high consistency between predicted and observed results. Decision curve analysis demonstrated favorable net benefits of the model.

**Conclusion:**

The predictive model established in this study holds promise for identifying arteriosclerosis in middle-aged and elderly individuals. Understanding the related risk factors and individualized prediction may assist physicians in early detection and intervention, ultimately improving patient prognosis.

## 1 Introduction

Adult mortality from cardiovascular disease is still the greatest cause of death, resulting in approximately 18 million deaths in 2019, and constituting 32% of the global mortality (1). It poses a significant worldwide public health challenge, emphasizing the importance of early identification and intervention for at-risk patients. Arteriosclerosis, involving elastin fiber fragmentation, collagen deposition, and arterial wall calcification, leads to reduced vascular tone and compliance. It serves as a marker of vascular aging (2), and is closely linked to cardiovascular disease (3), all-cause mortality (4), and cognitive decline (5). It is worth noting that it is different from the term “atherosclerosis.” Atherosclerosis refers to the narrowing of arteries due to plaque deposition. It is an inflammatory disease and also the main cause of cardiovascular disease and stroke (6).

When assessing arterial stiffness, pulse wave velocity (PWV) is the most reliable measurement (7), in particular, brachial-ankle pulse wave velocity (baPWV), is widely utilized to assess vascular injury in the general Asian population due to its convenience (8, 9), values of baPWV have a favorable correlation with the risk of hypertension and cardiovascular disease (10).

However, PWV testing is not extensively performed in primary community healthcare settings, and methods for assessing arteriosclerosis are limited. Identifying the risk factors for arteriosclerosis might have a significant therapeutic impact before clinical arteriosclerosis develops and progresses. A nomogram can predict outcome event probabilities based on subject characteristics and display the impact of each variable on the outcome, serving as a convenient tool for individualized disease diagnosis, assessment, and prognosis prediction (11, 12). Thus, this research aimed to create prediction models and investigate the factors that influence arteriosclerosis in community members.

## 2 Materials and methods

### 2.1 Study population

As part of the REACTION study (13), residents older than 40 years were enrolled in the first standardized questionnaire survey at baseline between December 2011 and April 2012. The follow-up survey was conducted from April 2015 to October 2015 over a mean duration of 3.25 ± 1.14 years. Exclusions included patients with arteriosclerosis in the first baseline survey, incomplete data, and loss to follow-up in the second on-site follow-up survey, resulting in a final sample of 4107 participants (Supplementary Figure 1). This study was approved by the Ethics Committee of the People’s Liberation Army General Hospital, and all study subjects signed informed consent. This study was carried out by the Declaration of Helsinki.

### 2.2 Data collection

Patient demographic information, living habits, past disease history and other basic information of the participants were collected by professionals through standardized questionnaires, and anthropometric parameters were measured by uniformly trained professionals. Height was measured with participants barefoot. Weight was measured with participants in light clothing. Before blood pressure measurement, a minimum of three measures were obtained, and participants were guaranteed to remain still for over 10 min. Waist circumference was obtained using a medical tape measure around the umbilical cord. Blood and urine samples were taken the next morning following a fast after dinner in order to test biochemical indicators such as uric acid, creatinine, low-density lipoprotein cholesterol (LDL), and glycosylated hemoglobin (HbA1c). Professionals used an Omron automated arteriosclerosis diagnostic device to measure baPWV. A cuff was knotted to the brachial artery of each upper arm and 2 cm above the medial malleolar of each lower limb after the patient had been still for 3 min. The left and right sides’ baPWV values were automatically measured by the device, and the average value of the two sides was used for analysis throughout the investigation. The study only collected baPWV values and did not record the ankle-brachial pulse waveform morphology, as the research focused primarily on arterial stiffness rather than peripheral vascular hemodynamic morphology.

### 2.3 Definition of variables

Weight (kg) divided by height (m^2^) squared was used to compute body mass index (BMI). Drinking habits were classified as no drinking, occasional drinking, or regular drinking. Smoking habits were defined as no smoking, occasional smoking, or regular smoking. The estimated glomerular filtration rate (eGFR) was calculated as 186 × [serum creatinine (mmol/L)/88.41]^–1.154^ × (age)^–0.203^, eGFR of <60 mL/min/1.73 m^2^ is defined as chronic kidney disease (CKD) (14). The primary outcome is incident arteriosclerosis, defined as baPWV ≥ 1400 cm/s (15, 16). This cutoff value is based on previous research evidence in East Asian populations and is highly consistent with the characteristics of the study population, ensuring the accuracy of diagnosis. Hypertension is defined as systolic blood pressure (SBP) ≥ 140 mm of mercury (mmHg), diastolic blood pressure (DBP) ≥ 90 mmHg, or current use of antihypertensive medications (17). The sex indicator refers to the biological sex of the respondents; the coding rule is defined as men = 0 and women = 1.

### 2.4 Statistical methods

For missing values of baseline variables, the Multiple Imputation by Chained Equations method was used for imputation. The single exclusion method was not adopted to reduce sample loss, and core variables such as age and systolic blood pressure were included as auxiliary variables during the imputation process to improve accuracy. To investigate and create a nomogram, factors associated with arteriosclerosis were evaluated using least absolute shrinkage and selection operator (LASSO) regression and multivariate logistic regression. When using LASSO regression for variable selection, the optimal λ value was determined through 10-fold cross-validation, which effectively alleviates multicollinearity; meanwhile, Bootstrap sampling (number of samplings = 1000) was used to evaluate the overfitting risk of the model. Decision curve analysis, calibration curves, and receiver operating characteristic (ROC) curves were used to evaluate the model’s goodness of fit, discriminating ability, and clinical application efficacy. A *P*-value < 0.05, indicated statistical significance was established. IBM SPSS Statistics 25 and R (4.1.2) were used for all the statistical analyses.

## 3 Results

### 3.1 Clinical features of research participants

The study enrolled 4107 middle-aged and elderly individuals, who were split into two cohorts–one for derivation (*n* = 2875) and one for validation (*n* = 1232)–for a 7:3 ratio. Table 1 presents the demographic characteristics and clinical data such as age, sex, smoking habits, aspartate transferase, and systolic blood pressure, of the participants in the two cohorts, no significant differences were found (*P* > 0.05).

**TABLE 1:** Demographic and clinical characteristics of the derivation and validation cohorts.

Variables	Derivation cohort	Validation cohort	*P*-value
*n*	2875	1232	
Age, years	60.53 ± 7.16	60.74 ± 7.08	0.213
Men, %	1035 (36.6%)	448 (36.4%)	0.161
BMI, kg/m^2^	25.15 ± 3.53	25.09 ± 3.29	0.612
WC, cm	86.20 (82.00, 90.00)	86.40 (82.00, 91.00)	0.326
SBP, mmHg	129.00 (118.00, 143.00)	128.00 (117.00, 142.00)	0.558
DBP, mmHg	78.00 (71.00, 86.00)	78.00 (71.00, 86.00)	0.522
TC, mmol/L	5.06 (4.27, 5.86)	5.06 (4.29, 5.87)	0.245
TG, mmol/L	1.63 (1.13, 2.34)	1.64 (1.08, 2.22)	0.432
HDL, mmol/L	1.21 (1.02, 1.42)	1.22 (1.04, 1.45)	0.347
LDL, mmol/L	2.89 (2.31, 3.56)	2.90 (2.34, 3.57)	0.463
FBG, mmol/L	5.16 (4.82, 5.67)	5.19 (4.80, 5.65)	0.235
PBG, mmol/L	7.46 (6.20, 9.50)	7.47 (6.22, 9.53)	0.321
HbA1c, %	5.70 ± 0.60	5.70 ± 0.60	0.711
UACR, mg/g	11.25 (6.47, 18.38)	11.28 (6.50, 18.40)	0.063
Creatinine, mg/dl	67.10 (60.20, 76.50)	67.10 (60.30, 76.40)	0.322
ALT, U/L	16.00 (12.00, 24.00)	16.00 (13.00, 24.00)	0.129
AST, U/L	20.00 (16.00, 26.00)	20.00 (17.00, 26.00)	0.319
GGT, U/L	20.00 (16.00, 25.00)	20.00 (16.00, 25.00)	0.260
eGFR, mL/min/1.73 m^2^	100.63 (92.64, 128.52)	100.51 (92.84, 127.64)	0.082
Working, %	561 (19.5%)	241 (19.6%)	0.917
Smoking habits, %			0.256
No	2435 (84.7%)	1041 (84.5%)	
Occasionally	78 (2.7%)	32 (2.6%)	
Usually	362 (12.6%)	159 (12.9%)	
Drinking habits, %			0.384
No	2196 (76.4%)	942 (76.5%)	
Occasionally	471 (16.4%)	205 (16.6%)	
Usually	208 (7.2%)	85 (6.9%)	
Exercise, %	865 (30.1%)	372 (30.2%)	0.159
Hypertension, %	526 (18.3%)	230 (18.6%)	0.354
CKD, %	293 (10.2%)	122 (9.9%)	0.103

Data expressed as mean ± SD for continuous variables or median (25 quantile, 75 quantile) for skewed variables and percentage (%) for categorical variables. BMI, body mass index; WC, waist circumstance; SBP, systolic blood pressure; DBP, diastolic blood pressure; TC, total cholesterol; TG, triglyceride; HDL, high-density lipoprotein cholesterol; LDL, low-density lipoprotein cholesterol; FBG, fasting blood glucose; PBG, 2-h postload blood glucose; HbA1c, glycosylated hemoglobin; ALT, alanine aminotransferase; AST, aspartate transferase; GGT, gamma-glutamyl transferase; UACR, urine albumin-to-creatinine ratio; eGFR, estimated glomerular filtration rate; CKD, chronic kidney disease.

Additionally, Table 2 outlines the baseline characteristics of the individuals in the derivation cohort stratified by baPWV, the arteriosclerosis group had substantially higher levels of age, BMI, usually smokers, fasting blood glucose, uric acid, total cholesterol, and other indicators in comparison to the normal group (*P* < 0.05).

**TABLE 2:** Demographic and clinical characteristics of derivation cohort by baPWV category.

Variables	baPWV < 1400 cm/s	baPWV ≥ 1400 cm/s	*P*-value
*n*	1690	1185	
Age, years	59.49 (54.65, 66.86)	62.58 (56.21, 70.66)	<0.001
Men, %	601 (35.6%)	498 (42.0%)	<0.001
BMI, kg/m^2^	25.01 ± 3.50	25.64 ± 3.45	<0.001
WC, cm	85.00 (79.00, 92.00)	87.00 (80.00, 94.00)	<0.001
SBP, mmHg	126.00 (117.00, 142.00)	136.00 (122.00, 154.00)	<0.001
DBP, mmHg	76.00 (70.00, 84.00)	81.00 (73.00, 89.00)	<0.001
TC, mmol/L	5.04 (4.27, 5.85)	5.10 (4.29, 5.89)	0.006
TG, mmol/L	1.59 (1.11, 2.29)	1.77 (1.21, 2.58)	<0.001
HDL, mmol/L	1.22 (1.03, 1.43)	1.18 (1.01, 1.39)	<0.001
LDL, mmol/L	2.86 (2.24, 3.51)	2.93 (2.33, 3.57)	<0.001
FBG, mmol/L	5.03 (4.86, 5.45)	5.34 (4.78, 6.20)	<0.001
PBG, mmol/L	7.40 (6.05, 9.07)	7.64 (6.80, 9.80)	<0.001
HbA1c, %	5.60 ± 0.60	5.90 ± 0.60	<0.001
UACR, mg/g	11.05 (6.38, 18.16)	11.97 (6.59, 19.18)	<0.001
Creatinine, mg/dl	66.70 (60.10, 75.30)	68.80 (61.00, 80.80)	<0.001
ALT, U/L	16.00 (12.00, 24.00)	17.00 (12.00, 25.00)	0.005
AST, U/L	20.00 (16.00, 25.00)	21.00 (17.00, 27.00)	0.008
GGT, U/L	20.00 (16.00, 25.00)	20.00 (16.00, 25.00)	0.364
eGFR, mL/min/1.73 m^2^	92.95 (89.68, 118.44)	96.13 (82.64, 112.52)	<0.001
Working, %	350 (20.7%)	211 (17.8%)	0.010
Smoking habits, %			<0.001
No	1463 (86.6%)	972 (82.0%)	0.002
Occasionally	45 (2.7%)	33 (2.8%)	
Usually	182 (10.7%)	180 (15.1%)	
Drinking habits, %			0.004
No	1304 (77.2%)	892 (75.3%)	
Occasionally	273 (16.2%)	198 (16.7%)	
Usually	113 (6.9%)	95 (8.0%)	
Exercise, %	540 (32.1%)	325 (27.4%)	<0.001

Data expressed as mean ± SD for continuous variables or median (25 quantile, 75 quantile) for skewed variables and percentage (%) for categorical variables. BMI, body mass index; WC, waist circumstance; SBP, systolic blood pressure; DBP, diastolic blood pressure; TC, total cholesterol; TG, triglyceride; HDL, high-density lipoprotein cholesterol; LDL, low-density lipoprotein cholesterol; FBG, fasting blood glucose; PBG, 2-h postload blood glucose; HbA1c, glycosylated hemoglobin; ALT, alanine aminotransferase; AST, aspartate transferase; GGT, gamma-glutamyl transferase; UACR, urine albumin-to-creatinine ratio; eGFR, estimated glomerular filtration rate.

### 3.2 Construction of the prediction model

**FIGURE 1:**
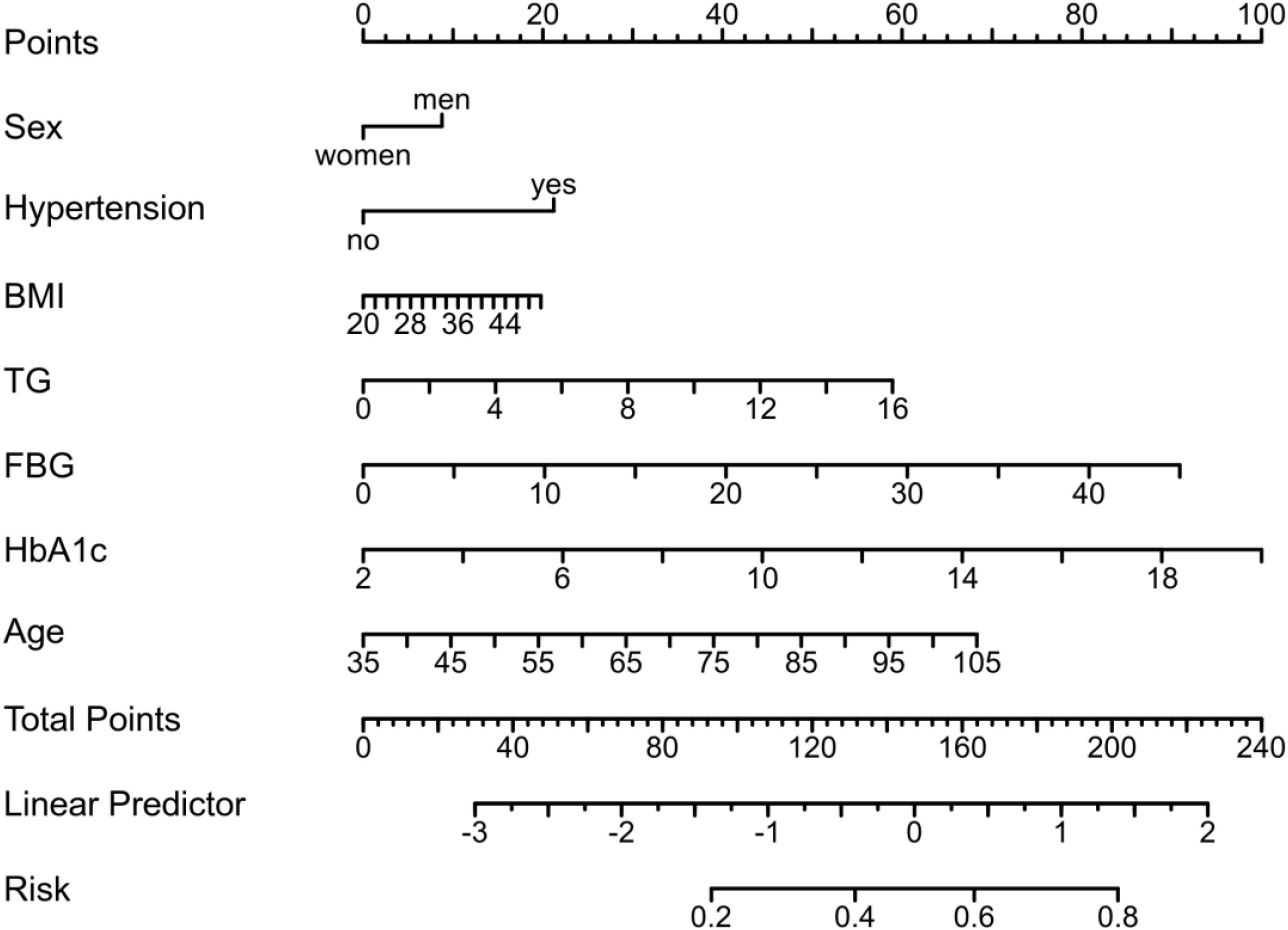
Nomogram for predicting arteriosclerosis. Each variable has a separate score, which is summed and then drawn by the vertical line to obtain the total score and the total risk.

By utilizing LASSO regression based on demographic and clinical data from the derivation cohort, potential prognostic factors were identified. Supplementary Figure 2A was used to determine the best penalty coefficient λ in the derivation cohort, and the topmost value corresponding to the dashed line on the left represents the number of variables included in constructing the optimal model. Supplementary Figure 2B shows the changes in the LASSO coefficient for all variables in the derivation cohort. Each line represents a variable. As the penalty term λ increases, the coefficient of all variables eventually becomes zero, but the rate of going to zero is inversely proportional to the variable importance. After the inclusion of 7 indicators, the optimal model was constructed–age, BMI, hypertension, triglycerides, glycated hemoglobin, sex, and fasting plasma glucose–with non-zero coefficients. Multivariate logistic analysis in Table 3 further confirmed the statistically significant relationship between these factors and arteriosclerosis, establishing them as independent risk factors. Compared with women, men are an independent risk factor for arteriosclerosis (OR = 1.269 [95% CI]: 1.143–1.408). Consequently, a nomogram was constructed (Figure 1), facilitating individual risk assessment for arteriosclerosis in middle-aged and elderly individuals.

**TABLE 3:** Multivariate logistic analysis of risk factors to arterial sclerosis in derivation cohort.

Variables	Odds ratio	95% CI	*P*-value
**Age**	1.054	1.026–1.084	0.003
**HbA1c**	1.148	1.096–1.202	<0.001
**FBG**	1.096	1.062–1.130	<0.001
**TG**	1.026	1.020–1.031	<0.001
**BMI**	1.012	1.009–1.015	<0.001
**Hypertension**			
No	Reference	1.459–1.784	<0.001
Yes	1.613		
**Sex**			
Women	Reference	1.143–1.408	0.001
Men	1.269		

HbA1c, glycosylated hemoglobin; FBG, fasting blood glucose; TG, triglyceride; BMI, body mass index.

### 3.3 Evaluation of the predictive model

**FIGURE 2:**
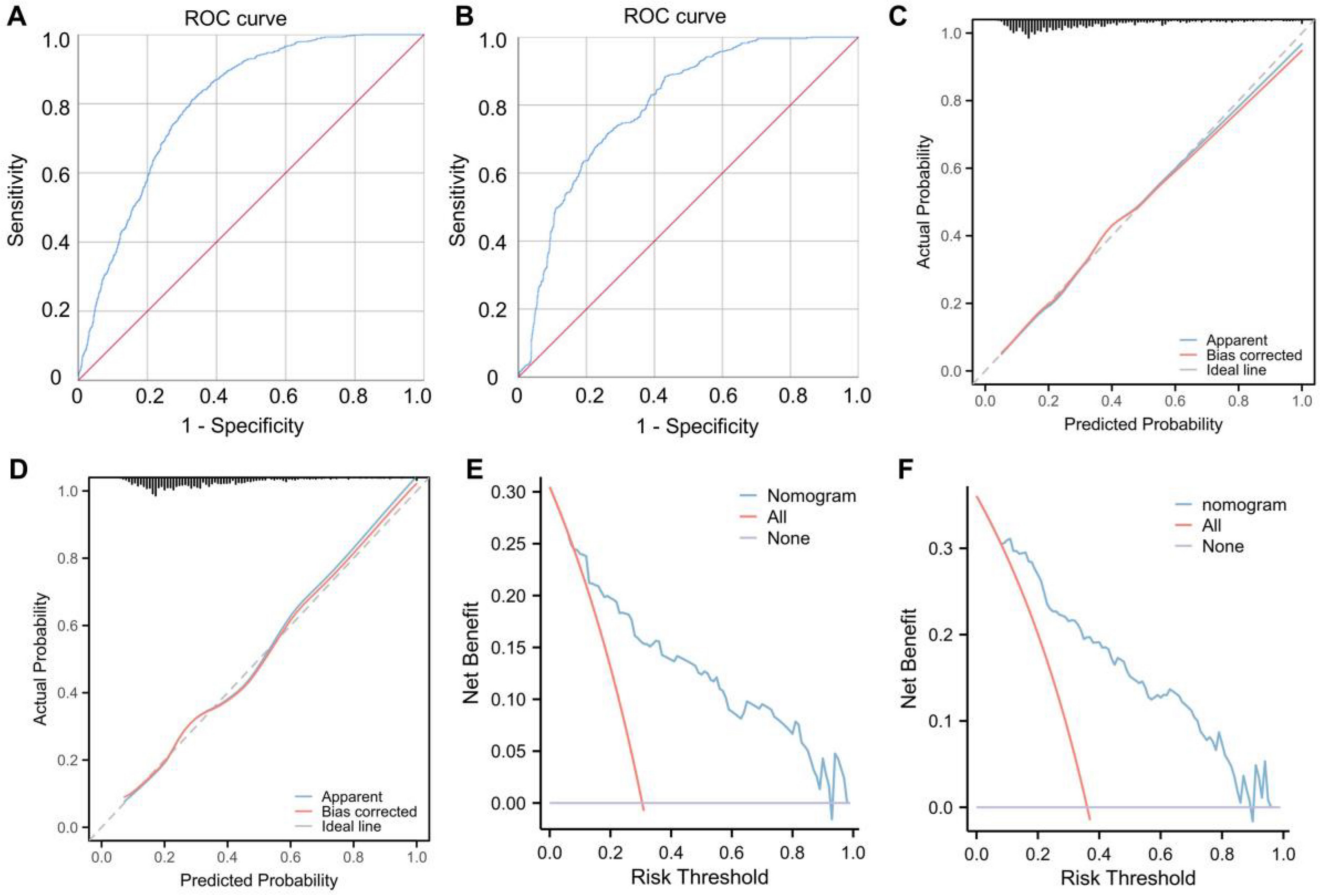
Area under the receiver operating characteristic (ROC) curve of the risk prediction model in the derivation cohort **(A)** and validation cohort **(B)**, calibration curves of the nomogram predictions in the derivation cohort **(C)** and validation cohort **(D)**, decision curve analysis of the nomogram predictions in the derivation cohort **(E)** and validation cohort **(F)**.

An area under the ROC curve (AUC) value of 0.811 (95% CI: 0.795–0.827) was obtained from the evaluation of the nomogram created from the derivation cohort (Figure 2A). After optimism correction using 1000 bootstrap samples, the corrected AUC in the derivation cohort was 0.796 (original AUC: 0.811), indicating good generalizability of the model. The validation cohort’s AUC value of 0.816 (95% CI: 0.796–0.830) was determined to evaluate clinical applicability and dependability (Figure 2B). With diagonal dashed lines representing the ideal state and blue lines representing the actual performance of the nomogram, high agreement was seen in the calibration curves between nomogram predictions and real data in the validation (Figure 2C) and derivation (Figure 2D) cohorts. The calibration plot shows that the predicted probabilities of the model are well-aligned with the actual occurrence probabilities, indicating no significant overestimation or underestimation of the predicted risk. The calibration effect is optimal especially in the moderate-to-high risk range (predicted risk: 60%–90%). The decision curve analysis curve shows that the model obtains good net benefits between threshold probabilities of 0.1 and 0.9, indicated both the derivation (Figure 2E) and validation (Figure 2F) cohorts showed high clinical practicability. The Hosmer-Lemeshow test, which revealed no discernible difference between expected and observed values, validated the model’s accuracy in both the derivation and validation cohorts.

## 4 Discussion

This cohort study focused on assessing the risk of arteriosclerosis in middle-aged and elderly individuals within the community, leading to the development of a nomogram for predicting arteriosclerosis risk. The investigation identified eight independent risk factors, including age, BMI, hypertension, triglycerides, glycosylated hemoglobin, sex, and fasting blood glucose. External validation demonstrated satisfactory results, indicating its potential as an efficient and convenient tool for identifying individuals at high risk of arteriosclerosis and guiding clinical decision-making.

Cardiovascular disease is a major public health challenge worldwide, which not only brings economic pressure but also reduces the quality of life. Arterial stiffness is a key link in cardiovascular diseases such as heart failure (3), atrial fibrillation (18), and is also closely related to vascular cognitive impairment, lacunar stroke, and chronic kidney disease (19). Therefore, early high-risk identification and intervention are necessary for the main prevention of arteriosclerosis. Previous studies that have explored predictive models for arterial stiffness have focused on the association of a single factor with arterial stiffness, such as hyperglycemia (20), hypertension (21), and physical activity (22) and have mostly been cross-sectional studies, which have limited power to determine the causal relationships between arterial stiffness and risk variables.

The pathogenesis of obesity-induced arterial stiffness may be related to increased vascular tone due to activation of the renin-angiotensin-aldosterone system (RAAS), oxidative stress, and insulin resistance (23). Microvascular and macrovascular changes induced by hyperglycemia and alterations in the extracellular matrix within the adventitia and elastic arterial media are thought to contribute to increased arterial stiffness in diabetic patients (24). Nicotine, a key component in cigarettes, elicits a systemic sympathomimetic response that leads to arterial stiffness and endothelial dysfunction (25). Elastic arteries’ structure and function are impacted by aging, which can also cause arterial stiffness by oxidative stress and systemic inflammation (26). High testosterone levels and low estrogen levels may account for the increased susceptibility of men to arterial stiffness (27).

Because of the increased relative risk among older persons, the aging population epidemic will result in a significant increase in the burden of disease related to arterial stiffness in the future (28). A growing body of research indicates that arterial stiffness can be altered by a variety of pharmacologic and lifestyle interventions. It has been demonstrated that weight loss (29), smoking cessation (30), aerobic exercise (31), and novel dietary interventions targeting the microbiome (32), among others, can all slow the advancement of arterial stiffness. In addition, treatment with medications that block the RAAS (33), and with statins (34) has been associated with reductions in measures of arterial stiffness.

The nomogram calculates the final score by adding the scores for each value level of each predictor based on the degree of influence. Ultimately, the link between the total score and the likelihood of result events allowed for the realization of the individual prediction of the probability of outcome events. This study developed a convenient and user-friendly nomogram for predicting arteriosclerosis. Its core function is as a risk stratification tool for arteriosclerosis in community populations, aiming to assist in targeted prevention by identifying high-risk individuals. Combined with clinical practice and the predictive distribution of the model, the risk thresholds are defined as follows: low risk < 30%, intermediate risk 30%–60%, and high risk > 60%. This tool can serve as an auxiliary means for community doctors to conduct first-line prevention and treatment of cardiovascular diseases, with two main application modes: First, the development of a simple web-based calculator, where community doctors input 7 indicators of patients (such as age, systolic blood pressure, etc.), and the calculator will automatically generate the predicted risk of arteriosclerosis and intervention recommendations. Second, integration with community electronic health record systems; when patients’ annual physical examination data are entered, the system will automatically invoke the nomogram for risk calculation, mark high-risk groups, and send follow-up reminders, thereby improving intervention efficiency. By re-evaluating baPWV annually to assess the effectiveness of intervention measures, this tool ultimately contributes to the primary prevention of cardiovascular diseases in the elderly population in China.

To the best of the knowledge, this is the first model built specifically for vascular stiffness in middle-aged and older community members. This study is not without limits, though. Firstly, only residents of community were included in the study, it was inevitably limited when extrapolating to other ethnic and national populations. Secondly, this is a cohort study, and there are inevitable biases such as loss to follow-up and confounding. Thirdly, carotid femoral pulse wave velocity (cfPWV) would have been a better option to quantify arterial stiffness, whereas there’s a strong positive connection (*r* = 0.73) between baPWV and cfPWV (34), and the American Heart Association suggests using baPWV to measure arterial stiffness (35). Fourthly, the outcome measurement time point of this study was 3.25 years after baseline, and the model did not conduct dynamic longitudinal prediction, which may not fully reflect the causal association between risk factors and arteriosclerosis. Fifthly, the study participants were recruited from communities, and most of the included individuals were those with strong health awareness who participated in regular physical examinations. This may lead to selection bias, resulting in reduced applicability of the model in populations with low health awareness. Sixthly, this study did not collect data on participants’ medication use history, daily dietary patterns, or inflammatory markers. However, these factors may affect arterial stiffness, and future studies should include them to improve the completeness of the model. Seventhly, this study only used an intra-cohort split (7:3 ratio) to construct derivation/validation cohort and did not perform independent validation with external datasets, which may limit the generalization ability of the model. Future studies will incorporate multicenter external data for further validation. Lastly, the relatively high incidence of incident arteriosclerosis observed in this study was mainly associated with the predominance of middle-aged and elderly participants and the high burden of vascular risk factors. Furthermore, this study only adopted a binary classification for baPWV, which could not rule out the possibility that some participants had baseline values close to the diagnostic threshold. This may lead to a “threshold-crossing” phenomenon and exert a certain impact on the event rate. Therefore, it is recommended that future studies retain continuous baPWV measurements to more accurately characterize the longitudinal progression of arterial stiffness.

## 5 Conclusion

Drawing from a substantial cohort study, this investigation identified risk factors linked to arteriosclerosis in individuals within the community. Additionally, it constructed a nomogram model with robust predictive capabilities. This visual tool serves dual purposes: firstly, aiding clinicians in identifying high-risk patients, thus facilitating early intervention to delay or potentially reverse disease progression. Secondly, it empowers patients to self-assess and improve their lifestyle habits, thereby deriving substantial benefits.

## Data Availability

The raw data supporting the conclusions of this article will be made available by the authors upon reasonable request.
