# Supplementary material for: Construction of a nomogram model to predict arteriosclerosis in middle-aged and elderly community dwellers: insights from a cohort study
Source: Front Med (Lausanne). 2026 Jun 19;13:1672197. doi: 10.3389/fmed.2026.1672197 (PMC13327931; doi:10.3389/fmed.2026.1672197)
Supplement: Supplementary file 1 [file Supplementary_file_1.docx]

**Supplementary figure 1** Flow chart of the study.


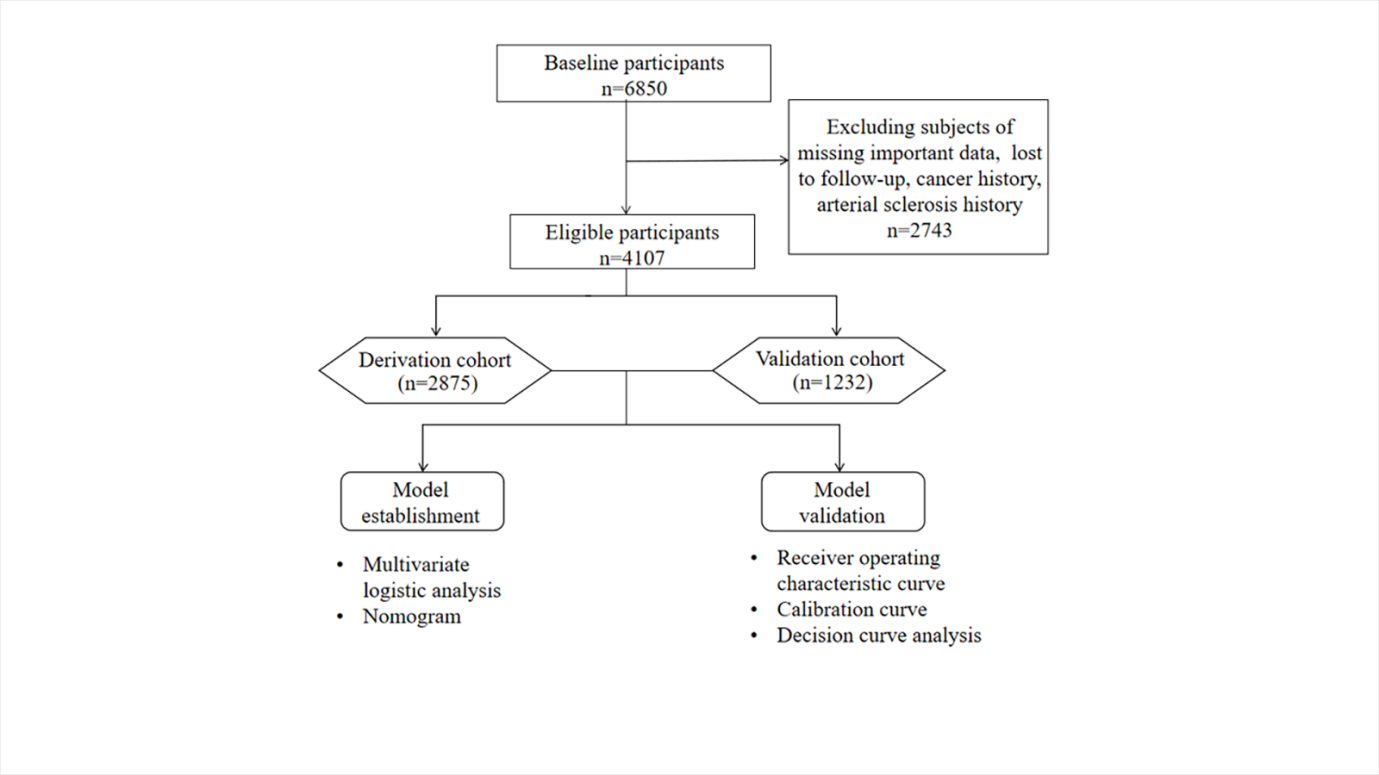


**Supplementary figure 2** (A) Least absolute shrinkage and selection operator (LASSO) binary logistic regression model. The optimal penalty coefficient λ was identified for the derivation cohort. (B) Changes in the LASSO coefficients for the seven variables in the derivation cohort.





**Supplementary figure 3 ROC analysis for different models for predicting arteriosclerosis.**


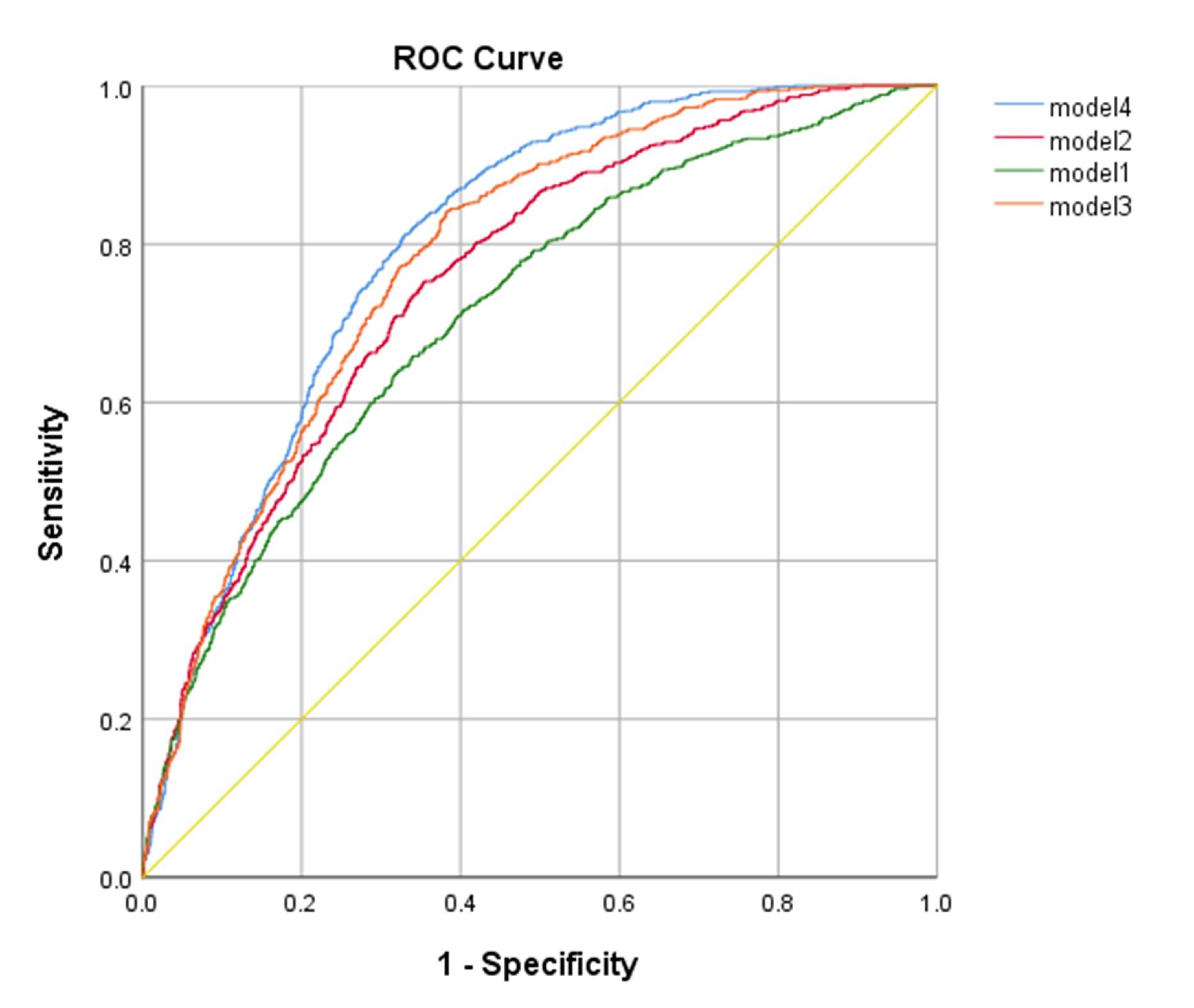


| Anthropometric Measures | AUC(95%CI) | *P* for difference in AUC |
| --- | --- | --- |
| Model 1 | 0.715 (0.693,0.737) | Reference |
| Model 2 | 0.757 (0.737,0.776) | <0.001 |
| Model 3 | 0.784 (0.766,0.802) | <0.001 |
| Model 4 | 0.811 (0.795,0.827) | <0.001 |

Notes: model 1 included age and hypertension; model 2 further included BMI; model 3 further included sex and HbA1c, and model 4 further included FBG and TG.
